# Anti-erosive effect of calcium carbonate suspensions

**DOI:** 10.4317/jced.54994

**Published:** 2018-08-01

**Authors:** Priscila Scandiffio, Tais Mantilla, Flávia Amaral, Fabiana França, Roberta Basting, Cecilia Turssi

**Affiliations:** 1 DDS, São Leopoldo Mandic Institute and Dental Research Center, Rua José Rocha Junqueira, 13, Campinas, SP, Brazil; 2 DDS, MSc, PhD student, School of Dentistry, University of São Paulo, Av. Professor Lineu Prestes 2227, São Paulo, SP, Brazil; 3 DDS, MSc, PhD, Assistant Professor, São Leopoldo Mandic Institute and Dental Research Center, Rua José Rocha Junqueira, 13, Campinas, SP, Brazil

## Abstract

**Background:**

This study aimed to investigate the ability of different concentrations of calcium carbonate (CaCO3) suspensions to control enamel surface loss.

**Material and Methods:**

Seventy-five enamel slabs were embedded, ground and polished in a pneumatic grinder-polisher machine. Reference areas were created with UPVC tape and the specimens were randomly allocated into five groups (n = 15) for exposure to hydrochloric acid solution to simulate gastric juice (0.01 M, pH 2) for 2 minutes. The samples were then exposed to suspensions containing 0.0001, 0.001, 0.01 or 0.1 mmol/L CaCO3 for 1 minute. Artificial saliva was used as control. The samples were subjected to a total of five erosive cycles followed by treatment with CaCO3 suspension. Surface loss was measured (in µm) using optical profilometry.

**Results:**

One-way ANOVA (*p* = 0.009) and Tukey’s test showed a significant reduction in surface loss when compared to the group not exposed to CaCO3 (0.74, +/- 0.23 µm), and the 0.01 mmol/L (0.40; +/- 0.23 µm) and 0.1 mmol/L suspensions (0.37; +/- 0.26 µm).

**Conclusions:**

The lower concentrated suspensions were incapable of significantly reducing enamel surface loss. Rinsing with 0.01 and 0.1 mmol/L calcium carbonate suspensions was revealed as a potentially promising strategy to prevent enamel erosion.

** Key words:**Tooth erosion, gastric acid, calcium carbonate, prevention and control.

## Introduction

Despite the inhomogeneity in the incidence and prevalence of dental erosion (1), cross-sectional, prospective and meta-analytic studies (2-4) have shown that approximately 30% of the population may have signs of wear due to intrinsic and extrinsic acids of non-bacterial origin. This situation underscores the need to better understand the etiology and management of dental erosion.

A cause of severe erosive lesions is the presence of hydrochloric acid from gastric juice (5), which may reach the dental surface due to episodes of recurrent emesis (vomiting), regurgitation of gastric contents caused by gastro esophageal reflux disease, as a consequence of bariatric surgery, due to anorexia and/or bulimia, via chronic alcoholism, and during pregnancy (6-9).

Hydrochloric acid is completely ionizable, which provides an exaggerated fall in oral pH, approximately 2.0 (10). In the presence of such acid, saliva becomes subsaturated in relation to the hydroxyapatite and fluorapatite crystals (11). Subsequently, the tooth undergoes mineral dissolution via erosive wear until oral homeostasis is restored by the rinsing, diluting and buffering effects of saliva (12). During this time, it is important to minimize erosion by implementing strategies that both prevent its occurrence and control the progression of existent lesions (13).

It is well accepted that the most important approach to control dental erosion caused by hydrochloric acid is the treatment of underlying medical conditions (13). However, it is important to highlight that while the cause behind the recurrent episodes of emesis is addressed, a dental surgeon should be included within the multidisciplinary team in order to educate the patient, as well as to provide preventive and/or therapeutic measures to reduce the damage caused by the contact between hydrochloric acid and the teeth (14-16).

The use of neutralizing agents, which contribute to alkalization of acids present within the oral cavity following erosive challenges, is one of the suggested approaches for erosion control (13,15,17-19). With this concept in mind, it has been reported that following challenges provided by solutions that simulate gastric juices, antacids reduced the time needed for the salivary pH to return to baseline (14,20). *In vitro* (21) and *in situ* (22,23) studies on neutralizing capacity have shown that following an erosive episode with simulated gastric juice, the use of alkaline solutions or suspensions, such as sodium bicarbonate and aluminum or magnesium hydroxide, significantly decreased enamel wear.

Calcium carbonate mouthwash suspensions may also potentially control dental erosion due to their strong buffering capacity, and hence may be considered a neutralizing agent. When hydrochloric acid comes into contact with calcium carbonate, the following chemical reaction ensues: CaCO3 + 2HCl → CaCl2 + CO2 + H2O, which provides acid neutralization alongside the formation of byproducts.

The potential of calcium carbonate to counteract dental erosion has still not been investigated. In addition, the possibility of a dose-dependent relationship between the calcium carbonate suspension and dental erosion control, explained by a higher or lower base availability to react with the acidic component, remains to be elucidated. Therefore, the aim of the present study, therefore, was to investigate the capacity of calcium carbonate suspensions at varying concentrations on the control of enamel surface loss following simulated erosive challenges.

## Material and Methods

-Experimental design

**Figure 1:**
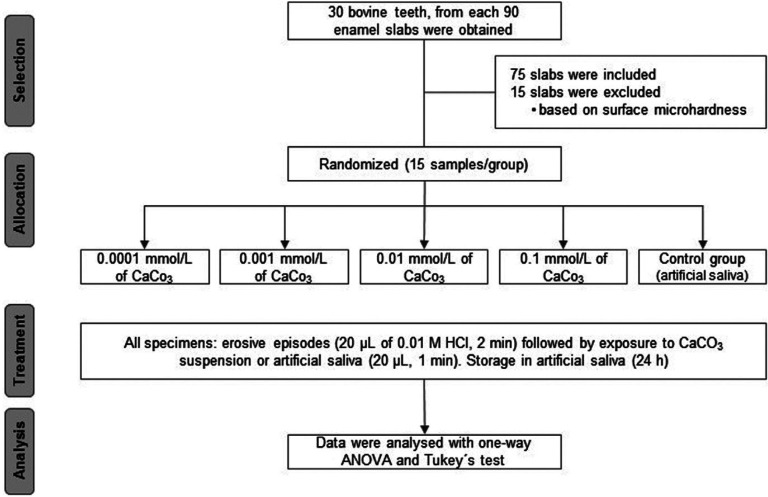
Flowchart of the experimental procedures.

A randomized complete block study with a unifactorial structure was designed. The study factor was Concentration of Calcium Carbonate Suspension at four experimental levels (0.0001 mmol/L; 0.001 mmol/L; 0.01 mmol/L; 0.1 mmol/L), as well as a control level, in which artificial saliva was used in place of the calcium carbonate suspension. Each group was composed of 15 test specimens (n = 15) obtained from slabs of bovine enamel. Sample size calculation, obtained using the GPower 3.1.9.2 software, was based on a pilot study with five samples per group, which suggested that for a one-way analysis of variance, an effect size of 0.48, and a significance level of 0.05, a total of 75 samples would be necessary to achieve a power of 90%. The response variable was surface loss, measured in µm. Figure 1 presents a flowchart of the experimental procedures in this study.

-Dental slab collection

Following approval by the Ethics Committee in Animal Research of the São Leopoldo Mandic Institute and Dental Research Center (#2013/0118), 30 bovine incisor teeth were obtained. Each tooth was cleaned with a scalpel blade, polished at low-speed with a pumice attached to a Robinson brush, and stored in 0.1% thymol solution.

In order to separate the roots from the crown, the teeth were cut with a precision saw (Isomet 1000, Buehler Ltd., Lake Bluff, IL, USA) at the dentin-enamel junction. Further cuts were made to the crown, providing 90 enamel slabs each measuring 3 x 3 x 2 mm (width x length x depth).

-Dental slabs planning and polishing

The dental slabs were embedded in epoxy resin (Epoxicure, Buehler Ltd., Lake Bluff, IL, USA), and ground wet with aluminum oxide papers (600 and 1200 grit) using a grinder-polisher machine equipped with a pneumatic system (Ecomet/Automet 250, Buehler Ltda., Lake Bluff, IL, USA). Polishing was achieved using felt discs and 0.3 µm alumina suspension (Alfa Micropolish, Buehler Ltd., Lake Bluff, IL, USA). Specimens were ultrasonically cleaned after each grinding and polishing step.

-Selection and preparation of specimens

Specimens were pre-tested using a HVS-1000 microhardness tester (Panambra Zwick Com. Máq. Equip. Ltda, São Paulo, SP, Brazil). Five Knoop microhardness indents (50 g, 15 s) were made in a linear fashion along the vertical center line, spaced 200 µm apart. The values obtained were submitted to descriptive analysis to obtain a frequency distribution curve, from which 75 test specimens were selected.

Adhesive unplasticised polyvinyl chloride (UPVC) tapes (Graphic Tape; Chartpak, Leeds, USA) were then placed along the right- and left-hand margins of the specimens leaving an exposed central area of 1 x 3 mm.

-Erosive episodes and application of calcium carbonate solutions

The specimens were subjected to erosive episodes using 20 µL of a 0.01 M hydrochloric acid solution (pH 2.0). Each sample was exposed to the acid for two minutes, after which the excess was removed.

Specimens were then arranged according to a random distribution into the control group (artificial saliva) or into one of the four experimental groups to be treated with of the following calcium carbonate suspensions – 0.0001 mmol/L; 0.001 mmol/L; 0.01 mmol/L; 0.1 mmol/L. The specimens (n = 15) were exposed to 20 µL of one of four calcium carbonate suspensions (pH 10) or artificial saliva for one minute after which they were rinsed with artificial saliva for 10 seconds and subsequently stored in artificial saliva for 24 hours at 37oC. Artificial saliva used comprised sodium hydroxymethylbenzoate, sodium carboxymethylcelullose, potassium chloride (KCl), MgCl2.6H2O, CaCl2.2H2O, K2HPO4 and KH2PO4 (24-26).

-Surface loss analysis

Surface loss measurements were performed using an optical profilometer (Proscan 2000, Scantron, Venture Way, Taunton, United Kingdom). In the x-axis, the step size and number of steps were set at 0.01 mm and 200, respectively; while, in the y-axis settings were 0.05 mm and 20, respectively. The accuracy on height measurements is 0.01 µm. The right and left lateral surfaces, which had been isolated with UPVC tape, were used as the reference areas. Images demonstrating the vertical loss formed between the reference areas and the regions submitted to treatment (µm) were analyzed using the Proscan Application software (version 2.0.17).

-Statistical analysis

Following descriptive analysis and confirmation of homogeneity of variance and normality, the data were subjected to inferential statistical analysis using one-way analysis of variance. Multiple comparisons were performed using the Tukey’s test. All statistical analyses were performed using the SPSS 20 software (SPSS Inc., Chicago, IL, USA), assuming a significance level of 5%.

## Results

**Figure 2:**
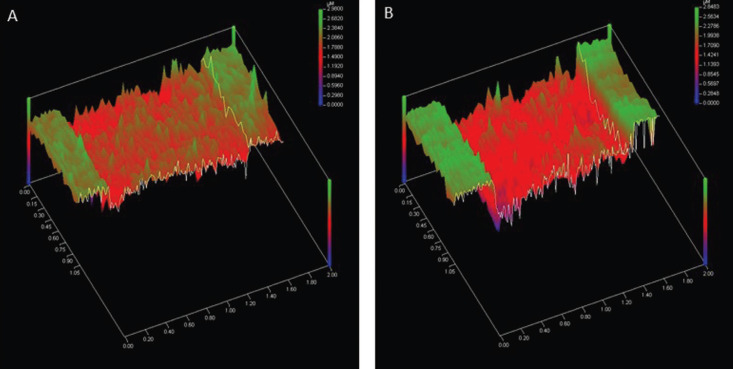
Profile corresponding to the scan area over the reference and worn areas of samples treated with 0.1 mmol/L CaCO3 suspension (A) and artificial saliva (B).

As confirmed by one-way analysis of variance, with a statistical power of 85.8%, surface loss of enamel following exposure to hydrochloric acid was affected by the use of calcium carbonate suspensions (*p* = 0.009). The Tukey’s test revealed that the enamel exposed to simulated gastric juices and then subjected to 0.01 and 0.1 mmol/L calcium carbonate suspensions showed a significantly decrease in surface loss when compared to the control group (artificial saliva). Figure 2 substantiates such difference. At concentrations of 0.0001 and 0.001 mmol/L, the calcium carbonate suspensions did not produce a significant effect when compared to control ( Table 1).

**Table 1:**
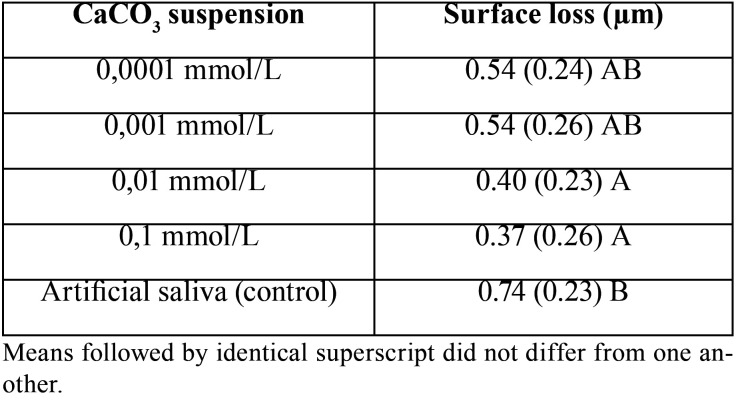
Net enamel loss according to the concentration of CaCO3 suspension used.

## Discussion

Despite the fact that saliva plays an important role in dental erosion, by rinsing, diluting and buffering both intrinsic and extrinsic acids (12), as well as allowing mineral deposition within these lesions (12), its capacity to prevent the formation and progression of dental erosions is limited. Therefore, new measures, such as those based on acid neutralization, have been adopted (21-23,26). With this in mind, the present study tested the hypothesis that calcium carbonate suspensions, in a dose-dependent manner, would have the ability to control enamel surface loss caused by hydrochloric acid, simulating gastric juices.

The results of this study confirmed the tested hypothesis, since wear caused by hydrochloric acid was significantly reduced depending on the concentration of calcium carbonate suspension used. These effect is based on the chemical reaction CaCO3 + 2HCl → CaCl2 + H2O + CO2, in which hydrochloric acid and calcium carbonate, the base component, form water, carbon dioxide and calcium chloride, therefore neutralizing the acid by consuming the H+ radicals. Consequently, an increase in pH of the dental surface is observed, and therefore a supersaturation in calcium and phosphate ions and decreased dissolution of hydroxyapatite and fluorapatite crystals are seen.

It is important to highlight that the effect of calcium carbonate suspensions is not restricted to dilution and rinsing of residual hydrochloric acid that remained on the enamel surface. This is proven by the fact that with artificial saliva (control group) a significantly inferior outcome was observed compared to the two highest concentrations of calcium carbonate used, which decreased surface loss by approximately 46 and 50% for the 0.01 and 0.1 mmol/L suspensions, respectively. It is worth speculating that like for fluoride mouthrinses, the protection exerted by such CaCO3 suspensions may depend on the number of erosive challenges (27). Under continuation of the erosive episodes, we speculate that the level of protection found for the two tested highly-concentrated CaCO3 suspensions might be reduced.

At lower calcium carbonate concentrations, 0.0001 and 0.001 mmol/L, the effect of the suspensions was not different from that of artificial saliva. This may be due to the fact that for the acid radical (H+) to be consumed in a way that impacts the control of enamel erosion there is a need of a minimum level of calcium carbonate, which was likely not offered by the 0.0001 and 0.001 mmol/L suspensions. Therefore, it can be stated the calcium carbonate suspensions tested in the present study exerted a concentration-dependent effect. One should reiterate, however, that there was no significant difference between the two lower concentration suspensions and the effect of artificial saliva.

The effect of neutralizing agents, such as the calcium carbonate used in this study, has been demonstrated for other suspensions. Sodium bicarbonate was used in the *in situ* model developed by Messias *et al.* (22), where the capacity to significantly reduce enamel wear by 27% was observed. Besides the fact that another experimental model was used in the quoted paper and may explain the difference between the efficacy of CaCO3 and sodium bicarbonate, it has been known since a long time ago that the equivalent power of CaCO3 is higher than that presented by sodium bicarbonate (28). Such property provides the former a stronger antacid power. In addition, in terms of commercially available products, sodium bicarbonate and magnesium hydroxide-based antacid suspensions, among others, have been highlighted as being capable of controlling erosion by up to 39% (21). A calcium carbonate suspension associated with sodium bicarbonate and alginate has also been shown to decrease enamel erosion by 38% (23).

At their higher concentrations (0.01 and 0.1 mmol/L), calcium carbonate suspensions have been shown to precipitate when left to rest. This is most likely due to the fact that at higher concentrations, the suspensions remains very close to its maximum value of solubility in water at 25ºC (0.12 mmol/L). In order to remove calcium carbonate residues that may have been present on the enamel slab surface, the test specimens were rinsed in an ultrasonic bath prior to being measured for surface loss. For such measurements we used proﬁlometry, which has been considered the gold standard method for measuring step height formation in in vitro erosion studies (29).

To improve ones understanding of the efficacy of calcium carbonate suspensions, one would need to investigate the presence of a time-dependent effect as well. In addition, it would be relevant to investigate whether CaCO3 formulations suspended in polymers would afford additional protection.

Although the effective management of the presence of gastric acid in the oral cavity demands the identification and treatment of the underlying pathology, the use of 0.01 and 0.1 mmol/L calcium carbonate mouthwashes seems a promising option to minimize the loss of the dental structure caused by hydrochloric acid. For that, further studies, including *in situ* and *in vivo* testing, are needed.
